# Quantitative Analysis of Adhesion Characteristics between Crumb Rubber Modified Asphalt and Aggregate Using Surface Free Energy Theory

**DOI:** 10.3390/ma15165735

**Published:** 2022-08-19

**Authors:** Ning Li, Jie Wang, Wei Si, Dongxia Hu

**Affiliations:** 1 School of Civil Engineering, Xi’an University of Architecture and Technology, Xi’an 710055, China; 2 Key Laboratory of Transport Industry of Road Structure and Material (Research Institute of Highway, Ministry of Transport), Beijing 100088, China; 3 Highway School, Chang’an University, Xi’an 710064, China; 4 School of Road and Bridge Engineering, Xinjiang Vocational and Technical College of Communication, Urumchi 831401, China

**Keywords:** crumb rubber modified asphalt, adhesion characteristics, surface free energy, adhesion work, spalling work, water stability

## Abstract

The utilization of waste rubber tires is of great value for environment protection and resource recovery, which can also improve the properties of matrix asphalt. The adhesion characteristics were evaluated for crumb rubber modified asphalt and limestone aggregate using the surface free energy (SFE) approach. Four types of matrix asphalt and four rubber contents were used to prepare the crumb rubber modified asphalt. The contact angle of matrix and crumb rubber modified asphalt was obtained, and the SFE indicators (dispersion, polar component, and compatibility rate—CR) were calculated. Moreover, the water stability tests were conducted using one matrix and rubber modified asphalt in order to investigate the relationship between SFE and water stability indicators. Results showed that the total SFE, dispersion component, adhesion work, and CR increased with the addition of crumb rubber, while the polar component and spalling work decreased. The types of asphalt had different influences on SFE indicators. The results from analysis of variation (ANOVA) indicated asphalt type and rubber content had significant influence on the adhesion work, spalling work and CR, and the influence of asphalt type was greater than that of rubber content. Additionally, the retained Marshall Stability and tensile strength ratio had better correlation with adhesion work and CR, but less with spalling work. The presented results demonstrated that the type of matrix asphalt played an important role in the adhesion characteristics for the crumb rubber modified asphalt.

## 1. Introduction

During the past few decades, numerous waste rubber tires were produced by the rapid growth in the number of automobiles, and their disposal became a significant challenge. Initially, direct landfilling and burning was the main treatment of waste rubber tires, which led to severe soil and air pollution [1,2]. Several approaches were put forward to solve this problem, such as tire retreading [3], thermal decomposition [4], pulverization into crumb rubber [1,5], etc. Among these approaches, using crumb rubber as asphalt modifier gained great attention. The crumb rubber modified asphalt mixture presented better mechanical properties than a matrix asphalt mixture, involving fatigue properties, crack resistance, rutting performance and aging resistance [1,5,6,7], etc. It provided an environmentally friendly method to efficiently utilize and recycle the waste rubber tires for the construction of asphalt pavement.

Numerous studies have been conducted to investigate the performance of crumb rubber modified asphalt and mixture. Generally, many factors would influence the properties of the crumb rubber modified asphalt mixture, including rubber content, particle size and desulfurization degree of crumb rubber [8], mixing temperature [9], matrix asphalt type [10,11], etc. Taking these factors into account, the properties of rubber modified asphalt were tested and evaluated, involving the physical properties (penetration, softening point, ductility, etc.), rheological properties (viscosity, phase angle, rutting resistance factor, complex modulus, etc.) and the chemical structure [6,12,13,14]. Simultaneously, the pavement performance of rubber modified asphalt mixture was also investigated, including high temperature, low temperature, water stability and fatigue resistance [10,15,16]. The indicators of asphalt and mixture were analyzed and compared, and then the optimal conditions were determined for the crumb rubber modified asphalt and mixture. In addition, the modification mechanism of rubber modified asphalt was explained from multi-scale tests, including the rheological tests, scanning electron microscope (SEM) tests, X-ray diffraction (XRD) tests, molecular dynamics simulation [6,17,18], etc. The investigations and discussions of the properties and modification mechanism greatly promoted the popularization and application of crumb rubber modified asphalt. Previous studies reported that the crack resistance and rutting performance obtained excellent evaluation for the crumb rubber modified asphalt mixture [6,10,16]. Except for the high and low temperature performance, water damage is also a significant concern in road performance [6,19]. However, limited work has been reported on the comparison of water stability considering the types of matrix asphalt of crumb rubber modified asphalt, as well as the rubber content. As for the crumb rubber modified asphalt mixture, some findings showed that the water stability can be improved to a certain extent by the wet process, but less significantly by the dry process [20,21,22]. In addition, some scholars stated that the influence on water stability is related to the type of aggregate and asphalt [23], while some scholars pointed out that the crumb rubber modified asphalt has little influence on the moisture resistance due to the lack of adhesion [10].

Adhesion characteristics between aggregate and asphalt critically affect the water stability and durability of asphalt mixture. For the poor adhesion of asphalt–aggregate composites, the common pavement distress (such as loose, peeling, alligator cracking, and potholes, etc.) appeared when water infiltrates into the mixture, even at the early stage of traffic service [14,20]. The stronger adhesion properties relates to some factors, such as asphalt and aggregate types, aggregate size and additives, etc. Bi et al. investigated the water stability of asphalt mixture using big data analyses considering the effect of asphalt and aggregate, pointing out that the asphalt manufacturers had little significant influence on water stability [24]. Niu et al. found that modified asphalt mixture with 5 wt% waste cooking oil and 20 wt% ground tire rubber displayed markedly improved water stability compared to normal asphalt mixture [25]. Traditionally, the water stability of asphalt mixture was evaluated and estimated using aggregate adhesion, immersion Marshall, freeze–thaw splitting and Hamburg rut tests [26,27], etc. These methods can effectively characterize the water stability when the various factors are considered. However, the influence mechanism was less addressed between the aggregate and rubber modified asphalt, and the adhesion characteristic was underrepresented.

Surface free energy (SFE) theory is a quantitative analysis approach that addresses the adhesion mechanism of asphalt mixture for water stability, which has been widely used to analyze the influence of different types of aggregate and asphalt and additive [28,29]. The adhesion work and spalling work of asphalt–aggregate composites was obtained based on SFE theory, as well as some evaluation indicators, such as compatibility rate (CR) [30], energy ratio (ER_1_ and ER_2_) [31] and comprehensive energy ratio (CER) [30,31,32], etc. The differences in indicators presented the obvious compatibility relationships for the various asphalt–aggregate composites, which can be used to analyze and reveal the influence mechanism of water stability of the asphalt–aggregate composites. With the comparison of adhesion work and ER, Hu et al. evaluated the moisture susceptibility of asphalt mixtures caused by the effects of foamed/modified asphalt and aggregate type [30]. The combination of asphalt binder and aggregates was recommended in order to improve the resistance of moisture stability and avoid potential moisture-induced damage. Using the SFE approach and SEM analyses, Wang et al. discussed the adhesion of asphalt–aggregate composites before and after aging. They pointed out that the adhesion and water damage resistance of asphalt–aggregate composites decreased after aging and the decline trend from the pressure aging vessel test was more obvious than from the rolling thin-film oven test, but the compatibility of asphalt–aggregate composites was hardly affected by the aging process [31]. Geng investigated the adhesion properties of asphalt and broken pebbles with different particle sizes based on the SFE theory [32]. These studies provided well-organized calculation and analyses methods for the use of the SFE approach to find out the energy variation in different conditions. However, when considering the type of matrix asphalt, few studies were conducted to investigate the adhesion ability of crumb rubber modified asphalt–aggregate composites using the SFE theory.

The purpose of this study is to evaluate the influence of matrix asphalt type and crumb rubber content on the adhesion characteristics using the SFE theory. The component of SFE was calculated via the measured contact angle. Then, the adhesion work, spalling work and compatibility rate were obtained and analyzed. In addition, an analysis of variation test was conducted to identify the significance of asphalt type and rubber content. Further, the water stability tests were carried out with one type of asphalt in order to investigate the relationship between water stability and SFE indicators; the correlation was analyzed and the water stability was evaluated.

## 2. Materials and Methods

### 2.1. Materials

#### 2.1.1. Matrix Asphalt

Four types of matrix asphalt were used in this study as base materials for the production of crumb rubber modified asphalt. They were Gaofu asphalt 70# (marked as GF-70), Qinhuangdao asphalt 70# (marked as QD-70), Shell asphalt 70# (marked as S-70) and Tipco asphalt 70# (marked as T-70), respectively. According to the regulations in the specification JTG E20-2011 [33], the main properties of the four matrix asphalts were tested and obtained as shown in Table 1. The properties of matrix asphalt confirmed to the requirements in the technical specification JT/T 798-2019 and JTG F40-2004 [34,35].

#### 2.1.2. Crumb Rubber Powder

The crumb rubber was 80 mesh produced by grinding the rubber part of waste tire tread in a Xi’an rubber powder processing plant. To active the crumb rubber, the ultrasonic desulphurization was conducted with 30% degree. The physical properties and chemical component of the crumb rubber were shown in Table 2 and Table 3, respectively.

#### 2.1.3. Crumb Rubber Modified Asphalt

According to the specifications in JT/T 798-2019 and previous research results [26,34], the preparation of crumb rubber modified asphalt was listed as follows: a high-speed shearing device was used to produce the crumb rubber modified asphalt. When the matrix asphalt was heated to 180 °C, the crumb rubber powder was slowly poured into the matrix asphalt. It should be noted that the crumb rubber powder should not be poured on the nozzle in order to avoid blocking the shearing device. In addition, a stirring rod was used for the stirring process. When the crumb rubber powder evenly dispersed in the matrix asphalt, the high-speed shearing device was started with a speed at 3000 rmp, and the shearing and reaction time was about 45 min. After that, the prepared crumb rubber asphalt was poured into a bucket and stored in a drying oven at 25 °C for further laboratory tests. According to the testing regulations, with 21% rubber content, the properties of the four crumb rubber modified asphalt were obtained, as shown in Table 4.

In order to investigate the influence of rubber content on the adhesion, four dosages of crumb rubber powder were added into the matrix asphalt. The dosages were 15%, 18%, 21% and 24%, respectively.

#### 2.1.4. Aggregate

The used aggregate was crushed limestone, which was shipped from a quarry in Tongchuan, Shaanxi province, China. According to the aggregate size, four types of aggregate were included: 10–15 mm, 5–10 mm, 3–5 mm and 0–3 mm, respectively. The bulk specific gravity of coarse aggregate was 2.414 g/cm^3^, the crushing value was 11.3%, the elongated particle content was 8.9% and the water abrasion was 0.35%.

#### 2.1.5. Composition of Asphalt Mixture

**Figure 1:**
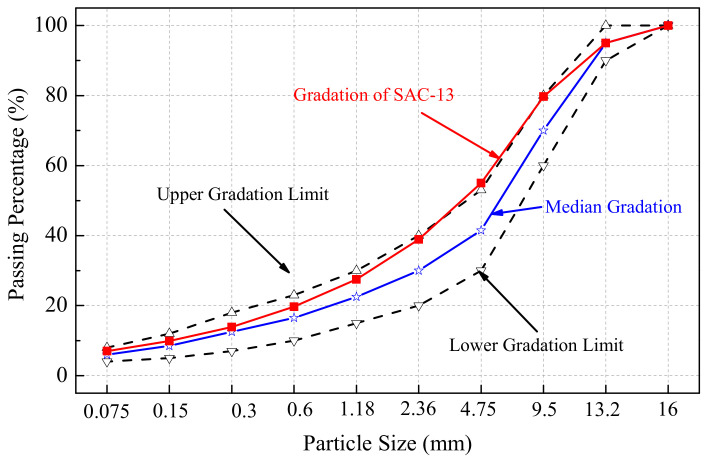
Aggregate gradation of SAC-13 mixture.

In this study, GF-70 matrix and modified asphalt was taken as the example for the water stability tests, in order to investigate the relationship between the SFE and water stability of the crumb rubber modified asphalt mixture. GF-70 matrix asphalt was used as the control sample, and the crumb rubber modified asphalt was regarded as the test group, which had rubber content at 15%, 18%, 21% and 24%, respectively. Then, five asphalt mixtures were used for the water stability tests. The type of asphalt mixture was SAC-13 (Stone Asphalt Concrete, in which the nominal maximum particle size of aggregate was 13.2 mm). The designed gradation of the aggregate was chosen following the specification of JTG/T F40-2004 [35] and literature [36], as shown in Figure 1. The optimum asphalt contents of the five mixtures were shown in Table 5.

### 2.2. Surface Free Energy (SFE) Theory

Surface free energy (SFE) refers to the increase in Gibbs function caused by the formation of a new per unit surface at a certain temperature and pressure, which is also equal to the required reversible work in the formation of a new per unit surface at a certain temperature and pressure. The SFE *γ* of a solid or liquid consists of the polar component *γ^p^* and the dispersion component *γ^d^*. As for the polar component *γ^p^*, it includes Lewis acid *γ*^+^ and Lewis base *γ*^−^, as presented in Equation (1).

(1) $$\gamma =\gamma^{d}+\gamma^{p}=\gamma^{d}+2\sqrt{\gamma^{-}\gamma^{+}}$$

For the solid–liquid interface, the dispersion force between the two interfaces can be expressed using the geometric mean of the dispersion components of the SFE of the liquid and solid, as well as the polar effect. Therefore, the SFE of liquid–solid interface *γ_sl_* can be expressed as Equation (2)

(2) $$\gamma_{sl}=\gamma_{s}+\gamma_{l}-2\sqrt{\gamma_{s}^{d}\gamma_{l}^{d}}-2\sqrt{\gamma_{s}^{p}\gamma_{l}^{p}}$$

in which *γ_sl_* represents the SFE of liquid–solid interface; *γ_s_* represents the SFE of the solid; *γ_l_* represents the SFE of liquid; *γ_s_^d^* represents the dispersion component of the SFE of the solid; *γ_l_^d^* represents the dispersion component of the SFE of the liquid; *γ_s_^p^* represents the polar component of the SFE of the solid; *γ_l_^p^* represents the polar component of the SFE of liquid.

This is combined with the Young Equation, as follows:

(3) $$\gamma_{s}=\gamma_{sl}+\gamma_{l}\cos\theta$$

In which *θ* is the contact angle between the interface. Then, Equation (2) can be converted into the following formation:

(4) $$\frac{1+\cos\theta }{2}\gamma_{l}=\sqrt{\gamma_{s}^{d}\gamma_{l}^{d}}+\sqrt{\gamma_{s}^{+}\gamma_{l}^{-}}+\sqrt{\gamma_{s}^{-}\gamma_{l}^{+}}$$

According to Equation (4), the surface energy parameters *γ_s_^d^* and *γ_s_^p^* of a solid can be obtained by measuring the contact angle (*θ*) of two known liquids with SFE parameters. Then, the SFE *γ_s_* of the solid was obtained using Equation (1).

### 2.3. Evaluation Indictor of SFE

In the mixture, asphalt wraps and adheres to the surface of aggregate, and then the SFE parameters of asphalt–aggregate composites will change. Without water, asphalt and aggregate forms a two-phase system, and the energy was released outwards. The adhesion work was proposed as the standard to evaluate the adhesion characteristic of solid and liquid, which refers to the released energy. The greater adhesion work means more energy released from the asphalt–aggregate composites. Then, the composites obtain the more stable state and the adhesion strength is greater. The adhesion work of asphalt–aggregate composites *W_as_* can be calculated by Equation (5).

(5) $$W_{as}=\gamma_{a}+\gamma_{s}-\gamma_{as}$$

In which *γ_a_* represents the SFE of asphalt; *γ_s_* represents the SFE of aggregate; *γ_as_* represents the SFE of asphalt–aggregate interface. Then, substituting Equation (2) into Equation (5), the adhesion work of asphalt–aggregate *W_as_* is presented in the following formation:

(6) $$W_{as}=2\sqrt{\gamma_{a}^{d}\gamma_{s}^{d}}+2\sqrt{\gamma_{a}^{+}\gamma_{s}^{-}}+2\sqrt{\gamma_{a}^{-}\gamma_{s}^{+}}$$

Considering the effect of water, the adhesion work of asphalt–water *W_aw_* and the adhesion work of aggregate–water *W_sw_* can be obtained using similar equations, as follows:

(7) $$W_{aw}=2\sqrt{\gamma_{a}^{d}\gamma_{w}^{d}}+2\sqrt{\gamma_{a}^{+}\gamma_{w}^{-}}+2\sqrt{\gamma_{a}^{-}\gamma_{w}^{+}}$$

(8) $$W_{sw}=2\sqrt{\gamma_{s}^{d}\gamma_{w}^{d}}+2\sqrt{\gamma_{s}^{+}\gamma_{w}^{-}}+2\sqrt{\gamma_{s}^{-}\gamma_{w}^{+}}$$

The adhesion work of asphalt–aggregate characterizes the adhesion capacity of asphalt and aggregate without water. Actually, the intrusion of water is hard to avoid, which usually leads to the asphalt–aggregate interface replaced by water–aggregate and water–asphalt interface. Then, the asphalt is stripped from the aggregate surface. Therefore, the spalling work *W_asw_* is used to evaluate the influence of water on the adhesion, which can be calculated using Equation (7):

(9) $$\begin{array}{l} W_{asw}=2\sqrt{\gamma_{a}^{d}\gamma_{w}^{d}}+2\sqrt{\gamma_{s}^{d}\gamma_{w}^{d}}-2\sqrt{\gamma_{a}^{d}\gamma_{s}^{d}}+2\sqrt{\gamma_{a}^{+}\gamma_{w}^{-}}+2\sqrt{\gamma_{s}^{+}\gamma_{w}^{-}}-\\ \;\;\;\;\;\;\;2\sqrt{\gamma_{a}^{+}\gamma_{s}^{-}}+2\sqrt{\gamma_{a}^{-}\gamma_{w}^{+}}+2\sqrt{\gamma_{s}^{+}\gamma_{w}^{-}}-2\sqrt{\gamma_{a}^{-}\gamma_{s}^{+}} \end{array}$$

As for the asphalt mixture, the greater spalling work *W_asw_* indicates worse resistance to water damage. Further, to illustrate the influence of adhesion, the compatibility rate *CR* was proposed in the previous studies [31,32], which was employed in this study to characterize the compatibility of adhesion between asphalt and aggregate. It was calculated using Equation (10).

(10) $$CR=\left|\frac{W_{as}}{W_{asw}}\right|$$

As presented in Equation (10), *W_as_* has a positive effect on the adhesion, while, *W_asw_* has a negative effect. Then, the greater compatibility rate *CR* contributed to stronger adhesion between asphalt and aggregate, and greater resistance to water damage.

### 2.4. Test Methods

#### 2.4.1. SFE Test of Crumb Rubber Modified Asphalt

The SFE parameters of the crumb rubber modified asphalt were measured using the lying drop method [30,32]. To obtain the parameters of SFE, distilled water, glycerol and formamide were used for asphalt. The first step is to prepare the crumb rubber modified asphalt film sheet. The specific operation procedures are as follows: we heated the crumb rubber modified asphalt to the melting state; immersed the glass sheet (the width was 2.5 mm) into the crumb rubber modified asphalt for 5 s and took it out; then, we hung it in the oven (120 °C for 30 min) so the excess asphalt could flow away and a smooth surface formed, avoiding bubbles; after that, we put the file sheet in the dryer oven for 4 h for the next step. Next, the optical contact angle device OCA20 was employed to obtain the contact angle between asphalt and distilled water, glycerol and formamide, respectively. The tests were carried out at 25 °C, each test had three parallel sheets, and eight measuring points were detected on each sheet. Thirdly, the contact angle was substituted into Equation (4) to create a regression equation group, as seen in Equation (11).

(11) $$\left[\begin{array}{l} \sqrt{\gamma_{w}^{d}}\;\;\;\sqrt{\gamma_{w}^{+}}\;\;\;\sqrt{\gamma_{w}^{-}}\\ \sqrt{\gamma_{g}^{d}}\;\;\;\sqrt{\gamma_{g}^{+}}\;\;\;\sqrt{\gamma_{g}^{-}}\\ \sqrt{\gamma_{f}^{d}}\;\;\;\sqrt{\gamma_{f}^{+}}\;\;\;\sqrt{\gamma_{f}^{-}} \end{array}\right]\;\left[\begin{array}{l} \sqrt{\gamma_{a}^{d}}\\ \sqrt{\gamma_{a}^{-}}\\ \sqrt{\gamma_{a}^{+}} \end{array}\right]=\frac{1}{2}\left[\begin{array}{l} \gamma_{w}(1+\cos(\theta_{w}))\\ \gamma_{g}(1+\cos(\theta_{g}))\\ \gamma_{f}(1+\cos(\theta_{f})) \end{array}\right]$$

in which *γ_w_^d^*, *γ_g_^d^*, *γ_f_^d^* represents the dispersion component of distilled water, glycerol and formamide, respectively; *γ_w_^+^*, *γ_g_^+^*, *γ_f_^+^* represents the Lewis acid of distilled water, glycerol and formamide, respectively; *γ_w_^−^*, *γ_g_^−^*, *γ_f_^−^* represents the Lewis base of distilled water, glycerol and formamide, respectively; *γ_a_^d^*, *γ_a_^−^*, *γ_a_^+^* represents the dispersion component, Lewis base and Lewis acid of the crumb rubber asphalt, respectively; *γ_w_*, *γ_g_*, *γ_f_* represents the SFE of distilled water, glycerol and formamide, respectively; *θ_w_*, *θ_f_*, *θ_e_* represents the contact angle between distilled water, glycerol, formamide and the crumb rubber asphalt, respectively. Then, the dispersion component *γ_a_^d^*, Lewis acid *γ_a_^+^* and Lewis base *γ_a_^−^* of the crumb rubber modified asphalt were obtained, and the polar component *γ_a_^p^*, and SFE *γ_a_* was calculated.

#### 2.4.2. SFE Parameters of Reagents

To obtain the contact angle of SFE, distilled water, glycerol and formamide were used for the crumb rubber modified asphalt, and distilled water, glycol and formamide were used for aggregate. Referred from literatures [28,31], the SFE parameters of the four reagents were shown in Table 6.

#### 2.4.3. SFE Test of Aggregate

To obtain the contact angle of limestone aggregate, the capillary rising method was used. Firstly, the aggregate was washed and dried. Then, the aggregate was crushed and put through a 0.15 mm sieve. Next, 3 g powder was weighed and put into a test tube and oscillated for 5 min [32]. The contact angle between powder and distilled water, glycol and formamide was measured using a dynamic contact angle measuring device and DCTA21 tensiometer. After that, the SFE of the limestone was calculated, as shown in Table 7.

#### 2.4.4. Water Stability Tests of Asphalt Mixture

According to the specifications in JTG E20-2011 [33], immersion Marshall and freeze–thaw splitting tests were conducted to evaluate the water stability of asphalt mixture. With the standard operations of immersion Marshall and freeze–thaw splitting tests, the retained Marshall Stability (*So*) and Tensile Strength Ratio (*TSR*) were obtained to represent the influence of water on the strength of an asphalt mixture, as shown in Equations (12) and (13). Each group test had four parallel specimens

(12) $$S_{0}=\frac{MS_{1}}{MS_{0}}\times 100\%$$

(13) $$TSR=\frac{\sigma_{2}}{\sigma_{1}}\times 100\%$$

in which *MS*_0_ represents the Marshall stability with 30 min immersion, kN; *MS*_1_ represents the Marshall stability with 48 h immersion, kN; σ_1_ represents the splitting strength at the normal condition, MPa; σ_2_ represents the splitting strength subjected to one freeze–thaw cycle, MPa.

## 3. Results

### 3.1. Contact Angle of Asphalt

**Figure 2:**
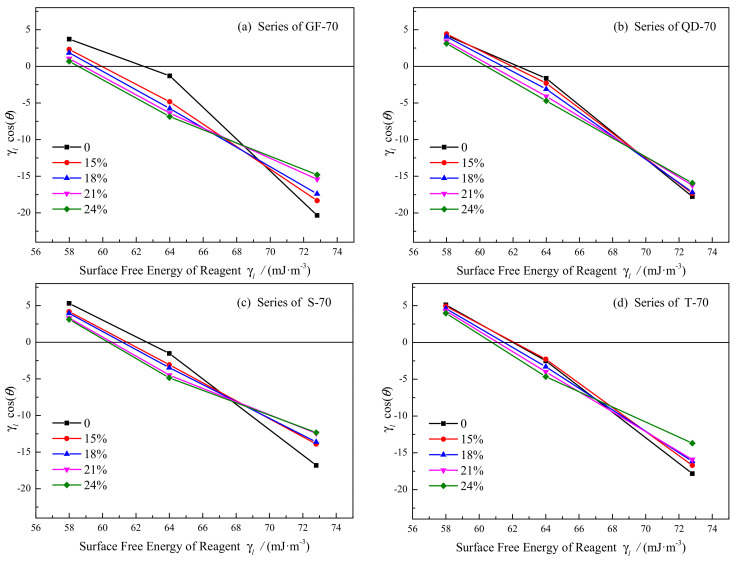
Relationship of *γ_l_* and *γ_l_*cos(*θ*) of the four series asphalt; (**a**) GF-70; (**b**) QD-70; (**c**) S-70; (**d**) T-70.

Using the lying drop method, the contact angle between asphalt and the three reagents was obtained. The average of the contact angle and the coefficient of variation (CV) was shown in Table 8. The value of CV was between 1.32% and 4.29%, which was much smaller than 5%. It indicated that the contact angle between asphalt and the reagents had great repeatability. In addition, the relationship between the SFE parameters of the reagents *γ_l_* and *γ_l_*cos(*θ*) was shown in Figure 2. The correlation coefficient (*R*^2^) of the linear regression ranged from 0.9080 to 0.9999 for the four asphalts, which revealed the linear relationship for *γ_l_* and *γ_l_*cos(*θ*) and verified the great effectiveness of the contact angle.

For the four types of asphalt, with the increase in the rubber content, the contact angle *θ_w_* decreased between distilled water and asphalt, while it increased for *θ_g_* and *θ_f_*. As for the four matrix asphalts, GF-70 had the greatest *θ_w_* and *θ_f_*, and S-70 had the smallest *θ_w_* and *θ_f_*. This showed that the hydrophilic and hydrophobic degree was different for the four matrix asphalts. GF-70 asphalt tended to be more hydrophobic than the other three matrix asphalts. When the crumb rubber was added, *θ_w_* decreased gradually, while *θ_g_* and *θ_f_* increased with the increase in the rubber content. When the rubber content increased from 15% to 24%, the variation in *θ_w_* was greater than the other two angles, and the variation in *θ_f_* was the smallest. The greatest variation range in *θ_w_* was up to 2.84° and 2.42° for GF-70 and T-70 asphalt, respectively, while the smallest variation range in *θ_f_* was 0.95° and 1.05° for T-70 and S-70 asphalt, respectively. It indicated that the hydrophobicity was enhanced for the crumb rubber modified asphalt. The greatest improvement was GF-70 asphalt. The light component of asphalt was absorbed by rubber during the swelling process, and the proportion of asphaltene and colloid increased. The light component was more likely to be hydrophilic. Less light component leads to improved hydrophobicity.

### 3.2. Surface Free Energy (SFE)

**Figure 3:**
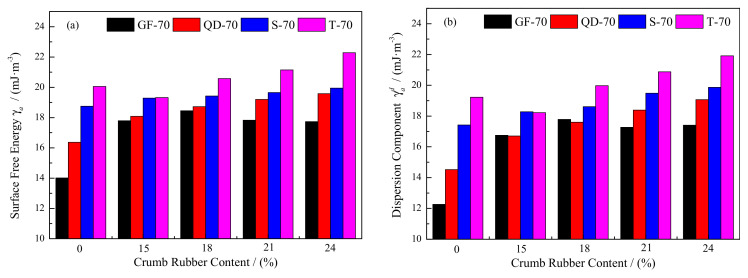

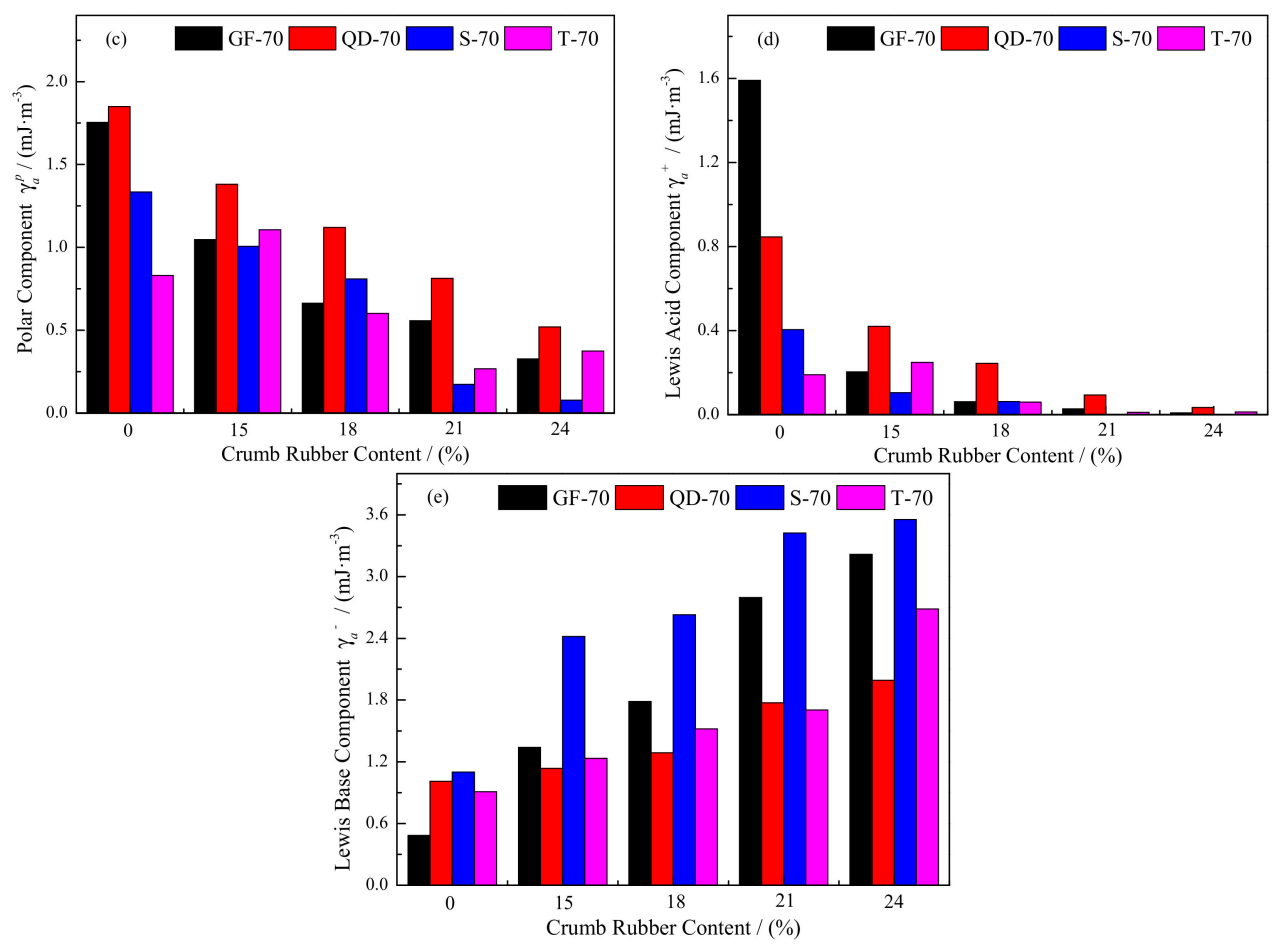
SFE of the matrix asphalt and crumb rubber asphalt; (**a**) total SFE *γ_a_*; (**b**) dispersion component *γ_a_^d^*; (**c**) polar component *γ_a_^p^*; (**d**) Lewis acid component *γ_a_^+^*; (**e**) Lewis base component *γ_a_^−^*.

According to the contact angle and Equation (11), the SFE parameters of the matrix asphalt and crumb rubber modified asphalt were obtained, as shown in Figure 3. With the increase in rubber content, the total SFE *γ_a_*, dispersion component *γ_a_^d^* and Lewis acid component *γ_a_^−^* increased, while the polar component *γ_a_^p^* and Lewis acid component *γ_a_^+^* decreased. Compared with the matrix asphalt, the maximum variation in *γ_a_*, *γ_a_^d^*, *γ_a_^p^*, *γ_a_^+^* and *γ_a_^−^* was 4.435, 5.525, 1.427, 1.582 and 2.732 mJ∙m^−^^2^, respectively. The minimum variation was 0.523, 0.752, 0.229, 0.058 and 0.125 mJ∙m^−^^2^, respectively. It indicated that *γ_a_^d^* was more obviously affected by crumb rubber. However, *γ_a_^+^* and *γ_a_^−^* obtained the most significant variation from the aspect of magnitude, and the average of variation was 83.3% and 173.8%, respectively. Although the variation magnitude of *γ_a_^+^* and *γ_a_^−^* was greater than that of *γ_a_*, *γ_a_^d^* and *γ_a_^p^*, its variation was smaller. The average of *γ_a_^d^* accounted for more than 95% of *γ_a_*, while *γ_a_^p^* accounted for less than 5%. It showed that the total SFE *γ_a_* of asphalt mainly depended on the dispersion component *γ_a_^d^*. As for *γ_a_^p^*, T-70 asphalt presented a different variation with the increase in rubber content. Subsequently, it first decreased and then increased. The lowest value was obtained at 21%.

As for the types of asphalt, the degree of influence of crumb rubber on the SFE was different. Among them, GF-70 asphalt obtained the greatest influence caused by the added crumb rubber, and the average variation in *γ_a_*, *γ_a_^d^* and *γ_a_^p^* was 28.1%, 41.1% and 63.0%. The total SFE of T-70 obtained the smallest influence caused by the crumb rubber. Moreover, the variation tendency of SFE parameters was not identical for the four types of asphalt. Compared with T-70 matrix asphalt, the crumb rubber modified asphalt had greater *γ_a_^p^* when the rubber content was 15%, while *γ_a_^p^* of the other modified asphalt was smaller than that of the matrix asphalt.

The variation indicated that the asphalt type and rubber content both affected the SFE of the modified asphalt. The differences in four components (resins, asphaltenes, saturates and aromatics) were the main reasons for the variation among the matrix asphalt. The light components (saturates and aromatics) were absorbed by the crumb rubber, and then the Lewis acid component *γ_a_^+^* went down, and the Lewis acid component *γ_a_^−^* went up.

### 3.3. Adhesion Work

**Figure 4:**
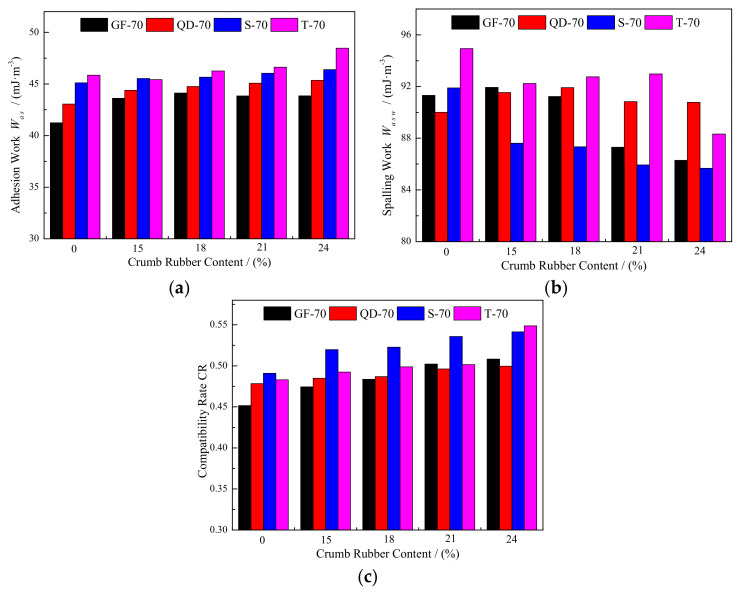
Adhesion work, spalling work and compatibility rate of the matrix asphalt and crumb rubber modified asphalt; (**a**) adhesion work *W_as_*; (**b**) spalling work *W_asw_*; (**c**) compatibility rate *CR*.

The adhesion work of the asphalt was calculated based on Equation (6), as shown in Figure 4a. For the matrix asphalt, T-70 had the greatest adhesion work and GF-70 obtained the smallest one. This indicated that the adhesion work was related to the type of matrix asphalt. The component of matrix asphalt was the main reason attributed to the differences. When crumb rubber was added, the adhesion work was improved, in which GF-70 was the greatest; the average increment was 2.62 mJ∙m^−^^2^ and the magnitude was 6.4%, while T-70 asphalt was the least affected; its average magnitude was only 1.8%. Different types of matrix asphalt presented various responses to the crumb rubber, as well as the adhesion work of crumb rubber modified asphalt.

With the increase in the rubber content, the adhesion work of QD-70, S-70 and T-70 asphalts increased, but it was parabolic variation for GF-70 asphalt. When the rubber content increased from 15% to 24%, the adhesion work of GF-70 varied only 0.506 mJ∙m^−^^2^. The greatest increment in adhesion work was 3.045 mJ∙m^−^^2^, which was obtained by T-70 modified asphalt, although the average increment in T-70 modified asphalt was the lowest one. This suggested that the crumb rubber content had the greatest influence on the adhesion work of T-70 modified asphalt, and smallest influence on GF-70 modified asphalt.

### 3.4. Spalling Work

When water was considered, the spalling work *W_asw_* was employed to evaluate the resistance to water damage of asphalt mixture. With Equation (9), the spalling work was calculated for the four types of asphalt, as shown in Figure 4b. As for the matrix asphalt, T-70 had the greatest spalling work and QD-70 obtained the smallest value. When the crumb rubber was added, the average variation of QD-70 modified asphalt increased, and it decreased for the other three types of modified asphalt compared with the matrix asphalt. The average variation in QD-70 modified asphalt was 1.257 mJ∙m^−^^2^, which accounted for 1.4% compared with the QD-70 matrix asphalt. The greatest and smallest reduction in spalling work was 5.247 and 2.127 mJ∙m^−^^2^, which accounted for 5.7% and 2.3% for S-70 and GF-70 asphalt, respectively. This indicated that the influence of crumb rubber was different for the four matrix asphalts. The added crumb rubber enhanced the resistance to spalling of GF-70, S-70 and T-70 matrix asphalt, but less for the spalling work of QD-70 asphalt.

When the rubber content increased from 15% to 24%, the spalling work of GF-70, QD-70 and S-70 modified asphalt decreased. It increased first and then decreased for T-70, but the entirety still presented a downward tendency. With the increase in rubber content, the greatest and smallest reduction in spalling work was 5.641 and 1.143 mJ∙m^−^^2^ for GF-70- and QD-70-modified asphalt, respectively. Although the spalling work of S-70 matrix asphalt was greatly influenced by the added crumb rubber, the influence in rubber content variation was small. In addition, the crumb rubber power had small influence on the spalling work of QD-70 matrix and modified asphalt.

### 3.5. Compatibility Rate

The compatibility rate (CR) of the four types of asphalt was calculated using Equation (10), as shown in Figure 4c. S-70 and GF-70 matrix asphalt obtained the greatest and smallest CR, respectively. When the crumb rubber was added, the average CR increased for the four types of asphalt. Compared with the matrix asphalt, the average increment of CR was 0.041, 0.014, 0.039 and 0.027 for GF-70, QD-70, S-70 and T-70 modified asphalt, respectively. Similar to the spalling work, the CR of QD-70 matrix asphalt was the least affected by the crumb rubber, and GF-70 and S-70 matrix asphalt were influenced the most. Based on the definition of CR, a greater CR means a stronger capability for water stability of the asphalt mixture. Therefore, the results showed that the crumb rubber can greatly improve the water-damage resistance of GF-70 and S-70 matrix asphalt.

With the increase in rubber content, CR presented a linear increase tendency for the four modified asphalts. The greatest and smallest increment was 0.056 and 0.015 for T-70- and QD-70-modified asphalt. This indicated that T-70-modified asphalt had the strongest water stability when mixed with the limestone aggregate, while the water stability of QD-70 modified asphalt was less influenced by the rubber content.

### 3.6. Water Stability of the Crumb Rubber Asphalt Mixture

**Figure 5:**
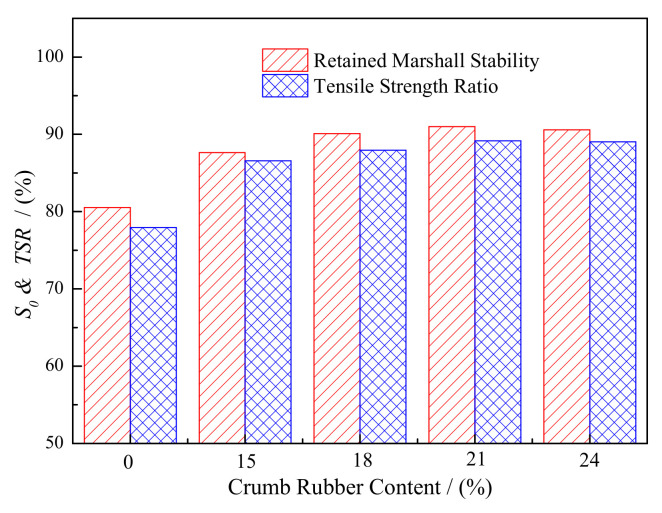
Retained Marshall Stability *S*_0_ and Tensile Strength Ratio *TSR* of GF-70 matrix and modified asphalt mixture.

The water stability tests were carried out for GF-70 matrix and modified asphalt mixture. The retained Marshall Stability (*S*_0_) and Tensile Strength Ratio (*TSR*) were calculated using Equations (12) and (13), as presented in Figure 5. This showed that *S*_0_ and *TSR* of the mixture increased when GF-70 matrix asphalt was modified by the crumb rubber. Compared with GF-70 matrix asphalt mixture, the average increment was 9.32% for *S*_0_ and 10.24% for *TSR*. This indicated that crumb rubber can improve the water stability of the asphalt mixture. On the other hand, *S*_0_ and *TSR* increased first and then decreased with the increase in rubber content. The peak point appeared at rubber content of 21%. The influence magnitude of *S*_0_ and *TSR* was 3.37% and 2.57%, respectively. It showed that the influence of crumb rubber on *S*_0_ was a little greater than that on *TSR*. Although the influence had some differences between *S*_0_ and *TSR*, the difference was small. From the view of SFE parameters, the adhesion work increased and the resistance of spalling was enhanced when the crumb rubber was added, and then the resistance to water damage was improved. This finding shows little difference from the previous results, in which the crumb rubber hardly affected the water stability of the asphalt mixture [10]. Two reasons were attributed to this difference: one was that the asphalt used was the matrix type without any additives; another was that the value of stability and tensile strength was relatively small for the used SAC-13 matrix asphalt mixture, although *S*_0_ and *TSR* was great. This indicated that the crumb rubber was more effective for the water stability of asphalt mixture which had lower stability and tensile strength.

## 4. Discussion

### 4.1. Analysis of Variance (ANOVA)

Two-factor ANOVA was carried out for *W_as_*, *W_asw_* and CR of the four types of asphalt, in which the asphalt type and rubber content were considered. The results were shown in Table 9. *SS* represents the sum of squares of deviation of the samples, *MS* represents mean square, *df* represents the degree of freedom, *F-test* represents the test statistics of F-distribution, *p*-value represents the test significance value and *F-crit* represents the critical statistics of F-distribution. When the *p*-value was greater than 0.05 and the *F-test* smaller than *F-crit*, the hypothesis (in which the rubber content had identical influence on the indicators) was accepted. Otherwise, it was determined that rubber content had significant influence on the indicators. The results from *F-test* and *p*-value showed that the asphalt type and rubber content both had significant influence on the indicator of *W_as_*, *W_asw_* and *CR*. The results were consistent with the aforementioned analyses and findings. Although the absolute value of the SFE and CR was small, the influence cannot be neglected for the two factors. The value of *F-test* and *p*-value showed that asphalt type had greater influence on the indicator than rubber content due to the greater *F-test* and smaller *p*-value of asphalt type. This finding emphasizes the importance of the asphalt type for the crumb rubber modified asphalt.

### 4.2. Correlation Analysis between SFE and Water Stability

Pearson correlation analysis was used to identify the relationship between SFE and water stability indicators of GF-70 matrix and modified asphalt mixtures. Results of correlation analysis for *W_as_*, *W_asw_*, CR, *S_0_* and *TSR* were tabulated in Table 10, which showed that *W_as_*, CR, *S_0_* and *TSR* had great correlation. Among them, the greatest correlation coefficient was up to 0.973, which was between *W_as_* and *S_0_*_._ In addition, *W_as_* presented quite good correlation with CR and *TSR*, and CR also had the better correlation coefficient with *S_0_* and *TSR*. The correlation coefficient between *S_0_* and *TSR* was 0.876, which verified the consistence of the water stability tests. However, the relationship between *W_asw_* and the other four indicators was relatively weak, especially for *W_as_* and CR. Combined with the results from ANOVA in Table 9, *F-test* value of *W_asw_* for the two factors was lower than that of *W_as_* and *CR*. This showed that the significance of asphalt type and rubber content reflected by *W_asw_* was weak compared with *W_as_* and CR. This indicated that *W_asw_* was not the best option to characterize the water stability. However, this finding was obtained for GF-70 asphalt mixture, and may be inappropriate for the other types of asphalt mixture.

In this study, the results showed that the adhesion properties of matrix asphalt were improved by the crumb rubber. The influence degree was different for the various types of matrix asphalt [37,38]. The main reason was related to the four components of the matrix asphalt. Generally, the light component in the matrix asphalt was more beneficial to the swelling modification of crumb rubber. Then, the binary modification was applied for the crumb rubber modified asphalt, which included waste cooking oil [25], gilsonite [27], waste engine oil [37] and other additives, etc. The crumb rubber modified asphalt can produce a three-dimensional network structure, and asphaltene suspended in the form of a gel [14]. This improved the bound force between the crumb rubber and asphaltene. Then, the composite turned into a stable structure, which contributed to the increase in water stability. In this aspect, the type of matrix asphalt plays a critical role in the modification of crumb rubber modified asphalt.

Contact angle also affects the accuracy and precision of the calculated energy and indicators. As presented in Table 8, the maximum difference in the contact angle reached up to 4.1°, although the CV was low (3.9%). However, the SFE parameters are sensitive to the variation in the contact angle, which leads to a difference in the SFE parameters. Considering the asphalt type and CV, the SFE showed some differences from other results [30,31,32].

On the other hand, the desulfurization degree is also a great factor for the modification effect, which is to make the crumb rubber hollow and cut off the carbon–sulfur and sulfur–sulfur bonds. Different desulfurization methods may cause a difference in the physical and chemical properties of crumb rubber [5,37,39]. Then, the used desulfurization method would be also a factor in the difference from the other results.

## 5. Conclusions

Based on the SFE theory, adhesion work, spalling work and compatibility rate were calculated and analyzed for the different asphalts, and water stability tests were also carried out with one type of asphalt mixture. The main conclusions were drawn as follows:

(1) The total SFE and dispersion component increased and the polar component decreased due to the added crumb rubber. As for the polar component of the crumb rubber modified asphalt, the Lewis acid component decreased, but the Lewis base component increased compared with the matrix asphalt.
(2) The influence of rubber content on the SFE parameters was different for the four matrix asphalts. For adhesion work, GF-70 obtained the greatest average increment, and T-70 had the smallest value caused by the added crumb rubber. However, with the increase in rubber content, the greatest variation in adhesion work was obtained by T-70, and the smallest was obtained by GF-70. GF-70- and QD-70-modified asphalt had the greatest and smallest reduction in spalling work. The spalling work of S-70 matrix asphalt was greatly influenced by the crumb rubber, but the influence of the rubber content was small
(3) As seen from CR, crumb rubber can greatly improve GF-70 and S-70 matrix asphalt’s resistance to water damage. T-70-modified asphalt had the strongest compatibility when mixed with the limestone aggregate. From the water stability tests of GF-70 asphalt mixture, *S*_0_ and *TSR* was improved when the crumb rubber was added. The greatest increment in *S*_0_ and *TSR* appeared at the rubber content of 21%.
(4) Asphalt type and rubber content both had significant influence on the adhesion work, spalling work and compatibility rate, and asphalt type had greater significance. Adhesion work and compatibility rate had better correlation with *S*_0_ and *TSR*, and it was lower for spalling work.

The obtained findings verified the effect of crumb rubber on the improvement of adhesion properties of asphalt mixture, as well as the rubber content. Moreover, they pointed out that the type of matrix asphalt has a significant influence on the modified asphalt. This is related to the four components of the matrix asphalt. The variation in four components for different asphalts (matrix asphalt and modified asphalt) is a good approach to find out the essential mechanism for relationship between the SFE and water stability indicators. In a future study, the relationship between the component and SFE will be investigated for asphalt. On the other hand, the used aggregate was only limestone which presented great variability due to its origins and ages. The calculated adhesion work, spalling work and compatibility rate presented some differences from the previous results.

## Figures and Tables

**Table 1:** Main properties of the four matrix asphalts.

Index	Test Value of Matrix Asphalt			
	GF-70	QD-70	S-70	T-70
Penetration at 25 °C/0.1 mm	67.6	68.8	72.4	65.2
Softening piont/°C	50.6	53.8	51.4	51.9
Ductility at 10 °C/cm	88.2	100.4	78.8	100
Viscosity at 135 °C/Pa∙s	0.532	0.714	0.433	0.485
Flash point/°C	263	267	269	262

**Table 2:** Physical properties of the crumb rubber.

Physical Index	Relative Density/kg∙m^−3^	Moisture Content/%	Metal Content/%	Fiber Content/%
Test value	1.18	0.35	0.006	0.54
Requirement	1.10~1.30	<1	<0.01	<1

**Table 3:** Chemical component of the crumb rubber.

Component	Rubber Hydrocarbon Content/%	Natural Rubber Content/%	Acetone Extract Content/%	Carbon Black Content/%	Ash Content/%
Test value	47.3	34.2	16.4	6.3	30.7
Requirement	≥42	≥30	≤22	≤8	≥28

**Table 4:** Properties of the crumb rubber modified asphalt (21% rubber content).

Index	Test Value of Crumb Rubber Modified Asphalt			
	GF-70	QD-70	S-70	T-70
Penetration at 25 °C/0.1 mm	38.6	36.8	43.4	44.2
Softening piont/°C	70.2	72.8	64.9	65
Ductility at 5 °C/cm	14.3	15.7	13.2	15.8
Viscosity at 180 °C/Pa∙s	4.617	5.484	3.578	4.292
Toughness/N∙m	13.4	12.8	14.6	15.3

**Table 5:** Optimum asphalt contents of the five mixtures.

Asphalt Type	Matrix	Rubber Content of Crumb Rubber Modified Asphalt			
	GF-70	15%	18%	21%	24%
Content (%)	4.8	5.2	5.4	5.5	5.7

**Table 6:** SFE parameters (mJ∙m^−^^2^) of the four reagents (at 25 °C).

Reagent	*γ_l_*	*γ_l_^d^*	*γ_l_^p^*	*γ_l_^+^*	*γ_l_^−^*
Distilled water	72.8	21.8	51.0	25.5	25.5
Glycerol	64.0	34.0	30.0	3.92	57.4
Formamide	58.0	39.0	19.0	2.28	39.6
Glycol	48.0	29.2	19.0	1.9	47.0

**Table 7:** SFE parameters (mJ∙m^−^^2^) of limestone (25 °C).

Aggregate	*γ_s_*	*γ_s_^d^*	*γ_s_^p^*	*γ_s_^+^*	*γ_s_^−^*
Limestone	28.04	22.43	5.61	1.16	6.79

**Table 8:** Contact angle between asphalt and the reagents (25 °C).

Asphalt Type	Rubber Content (%)	Distilled Water		Glycerol		Formamide	
		Average (°)	CV (%)	Average (°)	CV (%)	Average (°)	CV (%)
GF-70	0	106.23	2.53	91.17	1.76	86.34	2.09
	15	104.58	2.09	94.34	2.75	87.73	4.18
	18	103.84	3.08	95.17	2.09	88.19	2.42
	21	102.23	1.65	95.73	1.43	88.96	2.86
	24	101.74	1.87	96.16	1.43	89.34	1.65
QD-70	0	104.13	2.42	91.47	2.53	85.84	1.76
	15	103.77	3.96	92.06	2.97	85.63	3.63
	18	103.65	2.97	92.78	2.31	86.01	1.98
	21	102.88	2.09	93.67	1.87	86.62	3.96
	24	102.63	2.31	94.22	2.20	86.93	3.08
S-70	0	103.35	3.74	91.38	1.65	84.76	1.98
	15	101.01	2.31	92.76	1.98	85.88	3.41
	18	100.78	2.42	93.12	3.41	86.12	1.98
	21	99.83	3.41	94.03	2.09	86.74	4.29
	24	99.76	2.09	94.36	1.65	86.93	2.31
T-70	0	104.18	1.32	92.24	3.85	84.94	4.07
	15	103.26	1.54	92.04	1.87	85.12	2.31
	18	102.82	2.97	92.96	1.98	85.44	4.29
	21	102.63	2.09	93.58	2.20	85.75	1.87
	24	100.84	2.31	94.17	2.09	86.07	2.64

**Table 9:** Results of ANOVA.

Indicator	Factor	*SS*	*df*	*MS*	*F-Test*	*p*-Value	*F-Crit*
*W_as_*	Rubber content	10.800	4	2.700	7.167	0.003	3.259
	Asphalt type	29.408	3	9.803	26.021	0.000	3.490
	Error	4.521	12	0.377			
*W_asw_*	Rubber content	43.721	4	10.930	3.856	0.031	3.259
	Asphalt type	57.296	3	19.099	6.738	0.006	3.490
	Error	34.016	12	2.835			
*CR*	Rubber content	0.005	4	0.001	12.602	0.000	3.259
	Asphalt type	0.004	3	0.001	14.162	0.000	3.490
	Error	0.001	12	0.000			

**Table 10:** Pearson correlation coefficient for the indicators of GF-70 asphalt mixture.

Indicator	*W_as_*	*W_asw_*	*CR*	*S*_0_	*TSR*
*W_as_*	1				
*W_asw_*	0.494	1			
*CR*	0.918	0.108	1		
*S_0_*	0.973	0.395	0.932	1	
*TSR*	0.860	0.611	0.704	0.876	1

## Data Availability

The data presented in this study are available on request from the corresponding author. The data are not publicly available as they form part of an ongoing study.
